# Supplementary material for: Phytochemistry and Biological Studies of Endemic Hawaiian Plants
Source: Int J Mol Sci. 2023 Nov 14;24(22):16323. doi: 10.3390/ijms242216323 (PMC10670932; doi:10.3390/ijms242216323)
Supplement: Supplementary file 1 [file ijms-24-16323-s001.zip › ijms-2693899-supplementary.pdf]

Phytochemistry and biological studies of the endemic Hawaiian plants

Pornphimon Meesakul [1], Tyler Shea [2], Roland Fenstemacher [3], Shi Xuan Wong [4], Yutaka Kuroki [4], Aya Wada [4], Shugeng Cao [1,*]

[1] Department of Pharmaceutical Sciences, Daniel K. Inouye College of Pharmacy, University of Hawai'i at Hilo, 200 W. Kawili St., Hilo, HI 96720, USA; pmeesak@hawaii.edu
[2] Chemistry Department, University of Hawai'i at Hilo, 200 W. Kawili St., Hilo, HI 96720, USA; tylerms3@hawaii.edu
[3] Chemistry Laboratory, Board of Water Supply, City and County of Honolulu, 630 South Beretania Street, Honolulu, HI 96843, USA; hale_noa@yahoo.com
[4] Delightex Pte. Ltd., 230 Victoria Street, #15-01/08 Bugis Junction Towers, Singapore 188024, Singapore; shixuan@delightexplorers.com (S.X.W.); yutaka@delightexplorers.com (Y.K.); aya@delightexplorers.com (A.W.)
* Correspondence: scao@hawaii.edu

Table S1. The list of endemic Hawaiian plants has been reported.

No.	Benjamin C. Stone in 1967 [5]	Hawaiian Ethnobotany online database (Bishop Museum, 2023) [7]	The Hawaiian Islands website, National Museum of Natural History (NMNH) [6]	All endemic Hawaiian plants [5–7]
1	*Bidens cosmoides*	*Bidens amplectens*	*Argemone glauca* var. *glauca*	*Argemone glauca* var. *glauca*
2	*Charpentiera obovata*	*Bidens asymmetrica*	*Bidens amplectens*	*Bidens amplectens*
3	*Hillebrandia sandwicensis*	*Bidens campylotheca*	*Bidens asymmetrica*	*Bidens asymmetrica*
4	*Platydesma campanulatum, P. spathulatum, P. campanulata* (syn. *Melicope spathulata*)	*Bidens cervicata*	*Bidens campylotheca* subsp. *campylotheca*	*Bidens campylotheca*
5		*Bidens conjuncta*	*Bidens campylotheca* subsp. *pentamera*	*Bidens campylotheca* subsp. *campylotheca*
6		*Bidens cosmoides*	*Bidens campylotheca* subsp. *waihoiensis*	*Bidens campylotheca* subsp. *pentamera*
7		*Bidens forbesii*	*Bidens cervicata*	*Bidens campylotheca* subsp. *waihoiensis*
8		*Bidens hawaiensis*	*Bidens conjuncta*	*Bidens cervicata*
9		*Bidens hillebrandiana*	*Bidens cosmoides*	*Bidens conjuncta*
10		*Bidens macrocarpa*	*Bidens forbesii subsp. forbesii*	*Bidens cosmoides*
11		*Bidens mauiensis*	*Bidens forbesii subsp. kahiliensis*	*Bidens forbesii*
12		*Bidens menziesii*	*Bidens hawaiensis*	*Bidens forbesii* subsp. *forbesii*
13		*Bidens micrantha*	*Bidens hillebrandiana* subsp. *polycephala*	*Bidens forbesii* subsp. *kahiliensis*
14		*Bidens molokaiensis*	*Bidens macrocarpa*	*Bidens hawaiensis*
15		*Bidens populifolia*	*Bidens mauiensis*	*Bidens hillebrandiana*
16		*Bidens sandvicensis*	*Bidens menziesii* subsp. *filiformis*	*Bidens hillebrandiana* subsp. *polycephala*
17		*Bidens torta*	*Bidens menziesii* subsp. *menziesii*	*Bidens macrocarpa*
18		*Bidens valida*	*Bidens micrantha* subsp. *ctenophylla*	*Bidens mauiensis*
19		*Bidens wiebkei*	*Bidens micrantha* subsp. *kalealaha*	*Bidens menziesii*
20		*Charpentiera obovata*	*Bidens micrantha* subsp. *micrantha*	*Bidens menziesii* subsp. *filiformis*
21		*Clermontia persicifolia*	*Bidens molokaiensis*	*Bidens menziesii* subsp. *menziesii*
22		*Cuscuta sandwichiana*	*Bidens populifolia*	*Bidens micrantha*
23		*Dubautia arborea*	*Bidens sandvicensis* subsp. *confusa*	*Bidens micrantha* subsp. *ctenophylla*
24		*Gardenia brighamii*	*Bidens sandvicensis* subsp. *sandvicensis*	*Bidens micrantha* subsp. *kalealaha*
25		*Melicope barbigera (syn. Pelea barbigera)*	*Bidens torta*	*Bidens micrantha* subsp. *micrantha*
26		*Pipturus albidus*	*Bidens valida*	*Bidens molokaiensis*

No.	Benjamin C. Stone in 1967 [5]	Hawaiian Ethnobotany online database (Bishop Museum, 2023) [7]	The Hawaiian Islands website, National Museum of Natural History (NMNH) [6]	All endemic Hawaiian plants [5–7]
27		*Psychotria hawaiiensis*	*Bidens wiebkei*	*Bidens populifolia*
28		*Rauvolfia sandwicensis*	*Charpentiera obovata*	*Bidens sandvicensis*
29		*Santalum paniculatum*	*Clermontia persicifolia*	*Bidens sandvicensis* subsp. *confusa*
30		*Sophora chrysophylla*	*Coprosma ernodeides*	*Bidens sandvicensis* subsp. *sandvicensis*
31		*Vaccinium calycinum*	*Cuscuta sandwichiana*	*Bidens torta*
32		*Vaccinium reticulatum*	*Dryopteris mauiensis*	*Bidens valida*
33		*Wikstroemia monticola*	*Dubautia arborea*	*Bidens wiebkei*
34		*Wikstroemia uva-ursi*	*Erythrina sandwicensis*	*Charpentiera obovata*
35		*Zanthoxylum dipetalum*	*Hesperomannia arborescens*	*Clermontia persicifolia*
36		*Zanthoxylum hawaiiense*	*Hillebrandia sandwicensis*	*Coprosma ernodeides*
37		*Zanthoxylum kauaense*	*Lobelia yuccoides*	*Cuscuta sandwichiana*
38			*Lysimachia daphnoides*	*Dryopteris mauiensis*
39			*Lysimachia filifolia*	*Dubautia arborea*
40			*Lysimachia glutinosa*	*Erythrina sandwicensis*
41			*Lysimachia hillebrandii*	*Gardenia brighamii*
42			*Lysimachia iniki*	*Hesperomannia arborescens*
43			*Lysimachia kalalauensis*	*Hillebrandia sandwicensis*
44			*Lysimachia maxima*	*Lobelia yuccoides*
45			*Lysimachia ovoidea*	*Lysimachia daphnoides*
46			*Lysimachia pendens*	*Lysimachia filifolia*
47			*Lysimachia remyi*	*Lysimachia glutinosa*
48			*Lysimachia scopulensis*	*Lysimachia hillebrandii*
49			*Lysimachia waianaeensis*	*Lysimachia iniki*
50			*Phyllanthus distichus*	*Lysimachia kalalauensis*
51			*Pipturus albidus*	*Lysimachia maxima*
52			*Platydesma campanulatum, P. spathulatum, P. campanulata* (syn. *Melicope spathulata*)	*Lysimachia ovoidea*
53			*Rauvolfia sandwicensis*	*Lysimachia pendens*
54			*Sophora chrysophylla*	*Lysimachia remyi*
55			*Vaccinium calycinum*	*Lysimachia scopulensis*
56			*Vaccinium reticulatum*	*Lysimachia waianaeensis*

No.	Benjamin C. Stone in 1967 [5]	Hawaiian Ethnobotany online database (Bishop Museum, 2023) [7]	The Hawaiian Islands website, National Museum of Natural History (NMNH) [6]	All endemic Hawaiian plants [5–7]
57			*Wikstroemia monticola*	*Melicope barbigera* (syn. *Pelea barbigera*)
58			*Wilkesia gymnoxiphium*	*Phyllanthus distichus*
59			*Wilkesia hobdyi*	*Pipturus albidus*
60			*Zanthoxylum hawaiiense*	*Platydesma campanulatum, P. spathulatum, P. campanulata* (syn. *Melicope spathulata*)
61			*Zanthoxylum kauaense*	*Psychotria hawaiiensis*
62				*Rauvolfia sandwicensis*
63				*Santalum paniculatum*
64				*Sophora chrysophylla*
65				*Vaccinium calycinum*
66				*Vaccinium reticulatum*
67				*Wikstroemia monticola*
68				*Wikstroemia uva-ursi*
69				*Wilkesia gymnoxiphium*
70				*Wilkesia hobdyi*
71				*Zanthoxylum dipetalum*
72				*Zanthoxylum hawaiiense*
73				*Zanthoxylum kauaense*

Table S2. Phytochemistry and biological studies of the endemic Hawaiian plants.

No.	Plant	Parts used	Chemical components	Biological activity	Reference
1	*Argemone glauca* var. *glauca*	NA	Alkaloids	NA	[9]
2	*Bidens* genus				
	2.1 *Bidens amplectens*	Leaves, roots, flowers	Polyacetylenes, flavonoids	NA	[12,13]
	2.2 *Bidens asymmetrica*	Leaves, roots, flowers	Polyacetylenes, flavonoids	NA	[12,13]
	2.3 *Bidens campylotheca* subsp. *campylotheca*	Leaves, roots, flowers	Polyacetylenes, flavonoids	NA	[12,13]
	2.4 *Bidens campylotheca* subsp. *pentamera*	Leaves, roots, flowers	Polyacetylenes, flavonoids	NA	[12,13]
	2.5 *Bidens campylotheca* subsp. *waihoiensis*	Leaves and flowers	Flavonoids	NA	[13]
	2.6 *Bidens cervicata*	Leaves, roots, flowers	Polyacetylenes, flavonoids	NA	[12,13]
	2.7 *Bidens conjuncta*	Leaves, roots, flowers	Polyacetylenes, flavonoids	NA	[12,13]
	2.8 *Bidens cosmoides*	Leaves, roots, flowers	Polyacetylenes, flavonoids	NA	[12,13]
	2.9 *Bidens forbesii* subsp. *forbesii*	Leaves, roots, flowers	Polyacetylenes, flavonoids	NA	[12,13]
	2.10 *Bidens forbesii* subsp. *kahiliensis*	Leaves, roots, flowers	Polyacetylenes, flavonoids	NA	[12,13]
	2.11 *Bidens hawaiensis*	Leaves, roots, flowers	Polyacetylenes, flavonoids	NA	[12,13]
	2.12 *Bidens hillebrandiana*	Leaves and flowers	Flavonoids	NA	[13]
	2.13 *Bidens hillebrandiana* subsp. *polycephala*	Leaves and flowers	Flavonoids	NA	[13]
	2.14 *Bidens macrocarpa*	Leaves, roots, flowers	Polyacetylenes, flavonoids	NA	[12,13]
	2.15 *Bidens mauiensis*	Leaves, roots, flowers	Polyacetylenes, flavonoids	NA	[12,13]
	2.16 *Bidens menziesii* subsp. *filiformis*	Leaves, roots, flowers	Polyacetylenes, flavonoids	NA	[12,13]
	2.17 *Bidens menziesii* subsp. *menziesii*	Leaves, roots, flowers	Polyacetylenes, flavonoids	NA	[12,13]
	2.18 *Bidens micrantha* subsp. *ctenophylla*	Leaves, roots, flowers	Polyacetylenes, flavonoids	NA	[12,13]
	2.19 *Bidens micrantha* subsp. *kalealaha*	Leaves, roots, flowers	Polyacetylenes, flavonoids	NA	[12,13]
	2.20 *Bidens micrantha* subsp. *micrantha*	Leaves, roots, flowers	Polyacetylenes, flavonoids	NA	[12,13]
	2.21 *Bidens molokaiensis*	Leaves, roots, flowers	Polyacetylenes, flavonoids	NA	[12,13]
	2.22 *Bidens populifolia*	Leaves, roots, flowers	Polyacetylenes, flavonoids	NA	[12,13]

No.	Plant	Parts used	Chemical components	Biological activity	Reference
	2.23 *Bidens sandvicensis* subsp. *confusa*	Leaves, roots, flowers	Polyacetylenes, flavonoids	NA	[12,13]
	2.24 *Bidens sandvicensis* subsp. *sandvicensis*	Leaves, roots, flowers	Polyacetylenes, flavonoids	NA	[12,13]
	2.25 *Bidens torta*	Leaves, roots, flowers	Polyacetylenes, flavonoids	NA	[12,13]
	2.26 *Bidens valida*	Leaves, roots, flowers	Polyacetylenes, flavonoids	NA	[12,13]
	2.27 *Bidens wiebkei*	Leaves, roots, flowers	Polyacetylenes, flavonoids	NA	[12,13]
3	*Charpentiera obovata*	Roots, barks	Alkaloids	NA	[14,15]
4	*Clermontia persicifolia*	Leaves	Flavonoids	NA	[18]
5	*Coprosma ernodeoides*	Leaves, berries	Iridoid glycosides	Antioxidant properties	[19]
6	*Cuscuta sandwichiana*	NA	Macrocyclic glycoresins	Cytotoxicity	[21]
		Stems	NA	Antiviral activity against human immunodeficiency Virus Type-1 (HIV-1)	[25]
7	*Dryopteris mauiensis*	NA	Phenolics	NA	[28]
8	*Dubautia arborea*	Leaves	Flavonoids	NA	[29]
9	*Erythrina sandwicensis*	Seeds	Alkaloids	NA	[33–35]
		Fungus-inoculated leaves	Pterocarpans	NA	[36]
10	*Gardenia brighamii*	Leaves	NA	Antifungal activity against Fusarium species	[39]
11	*Hesperomannia arborescens*	Leaves	Flavonols, flavonoid glucosides	NA	[41]
12	*Hillebrandia sandwicensis*	Leaves	*C*-glycosyl flavones	NA	[42]
13	*Lobelia yuccoides*	Roots, stem barks	Alkaloids	NA	[43]
14	*Lysimachia* genus				
	14.1 *Lysimachia daphnoides*	Arial part	Flavonol glycosides	NA	[45]
	14.2 *Lysimachia filifolia*	Arial part	Flavonol glycosides	NA	[45]
	14.3 *Lysimachia glutinosa*	Arial part	Flavonol glycosides	NA	[45]
	14.4 *Lysimachia hillebrandii*	Arial part	Flavonol glycosides	NA	[45]
	14.5 *Lysimachia iniki*	Arial part	Flavonol glycosides	NA	[45]
	14.6 *Lysimachia kalalauensis*	Arial part	Flavonol glycosides	NA	[45]
	14.7 *Lysimachia maxima*	Arial part	Flavonol glycosides	NA	[45]
	14.8 *Lysimachia ovoidea*	Arial part	Flavonol glycosides	NA	[45]

No.	Plant	Parts used	Chemical components	Biological activity	Reference
	14.9 *Lysimachia pendens*	Arial part	Flavonol glycosides	NA	[45]
	14.10 *Lysimachia remyi*	Arial part	Flavonol glycosides	NA	[45]
	14.11 *Lysimachia scopulensis*	Arial part	Flavonol glycosides	NA	[45]
	14.12 *Lysimachia waianaeensis*	Arial part	Flavonol glycosides	NA	[45]
15	*Melicope barbigera* (syn. *Pelea barbigera*)	Leaves	Acetophenones, 2*H*-benzopyranes, isomeric melifoliones	Cytotoxic activities against the A2780 human ovarian cancer cell line	[48]
16	*Phyllanthus distichus*	Fruits	NA	Antimicrobial activity	[51]
		Fruits	Alkaloids, glycosides, phenolic compounds, reducing sugars, saponins, polyphenols, tannins, flavonoids, and terpenes	Antibacterial activity and antioxidant activities	[52]
		Leaves	NA	Hypoglycemic and antidiabetic properties	[53]
17	*Pipturus albidus*	Leaves	NA	Anti-microbial activity, anti-bacterial activity, and anti-fungal properties	[25]
		Leaves, bark, stems	NA	Antiviral activity	[57]
		Leaves	Macronutrients and minerals Phenolic acids	Antioxidant Activity	[54,61]
		Leaves	NA	Total antioxidant capacity	[61]
		Leaves	NA	Antioxidant, anticancer, and chemo preventive properties	[62]
		Leaves, bark, stems	Polyphenolics	Anti-viral, anti-fungal, anti-microbial, and anti-inflammatory attributes	[57]
		Leaves	NA	Antibacterial, antiviral properties, antioxidant effects, mild natural laxative properties, anti-allergic effects, promoting cardiovascular and liver health, and reducing stress levels	[58]
18	*Platydesma spathulatum or P. campanulatum or P. campanulata* (syn. *Melicope spathulata*)	Roots, stem barks, leaves	Alkaloids, furoquinolines	NA	[64]
19	*Psychotria hawaiiensis*	Barks, leaves	NA	Anti-viral against Herpes Simplex Virus 1 and 2A, anti-fungal, and anti-bacterial activities	[54]

No.	Plant	Parts used	Chemical components	Biological activity	Reference
		Barks, leaves	NA	Antiviral activity against human immunodeficiency virus type-1 (HIV-1)	[25]
20	*Rauvolfia sandwicensis*	Roots	Alkaloids	NA	[68,69]
21	*Santalum paniculatum*	Woods	Essential oils (terpenoids)	NA	[72]
		Leaves	NA	Antimicrobial activity against *Staphylococcus aureus*	[73]
		NA	Essential oils (terpenoids)	NA	[74]
		NA	Essential oils (terpenoids)	Potential bioactivity against *Tribolium castaneum* (red flour beetle)	[75]
22	*Sophora chrysophylla*	Barks	Quinolizidine alkaloids	NA	[76]
		Leaves, stems, seeds	Lupin alkaloids	NA	[77]
		Roots	Isoflavones and 6a-hydroxypterocarpans	NA	[78]
23	*Vaccinium* genus				
	23.1 *Vaccinium calycinum*	Leaves	Flavonoid glycosides, cinnamic ester	NA	[81]
		Fruits	NA	Antioxidant activity	[82]
		Fruits	NA	Antimicrobial activity against *Listeria monocytogenes*	[83]
		Fruits	Phenolics and anthocyanins	Antimicrobial activity against *Listeria monocytogenes* and *E. coli*	[84]
	23.2 *Vaccinium reticulatum*	Leaves	Flavonoid glycosides, cinnamic ester	NA	[81]
		Fruits	NA	Antioxidant activity	[82]
24	*Wikstroemia* genus				
	24.1 *Wikstroemia monticola*	Woody stems, barks	Daphnane diterpenes	Antitumor activity	[86–88]
	24.2 *Wikstroemia uva-ursi*	Whole plants	Lignans	Antitumor activity against the P-388 lymphocytic leukemia (3PS)	[86,94]
25	*Wilkesia* genus				
	25.1 *Wilkesia gymnoxiphium*	Leaves	Flavonoids (minor quantitative)	NA	[99]
	25.2 *Wilkesia hobdyi*	Leaves	Flavonoids (minor quantitative)	NA	[99]
26	*Zanthoxylum* genus				

No.	Plant	Parts used	Chemical components	Biological activity	Reference
	26.1 *Zanthoxylum dipetalum*	Root barks, root woods	Alkaloids, pyranocoumarins, triterpene, and flavonoid	NA	[101]
		Root barks	Pyranocoumarin and dipyranocoumarin	NA	[102]
		Leaves	Acyl histamines and protopine-type alkaloid	NA	[103]
		Leaves, pericarps	Volatile compounds	Insecticidal activity (ovicidal properties)	[105,106]
	26.2 *Zanthoxylum hawaiiense*	Leaves, pericarps	Volatile compounds	Insecticidal activity (ovicidal properties)	[105,106]
	26.3 *Zanthoxylum kauaense*	Leaves, pericarps	Volatile compounds	Insecticidal activity (ovicidal properties)	[105,106]

NA= Not available
